# Loot box engagement: A scoping review of primary studies on prevalence and association with problematic gaming and gambling

**DOI:** 10.1371/journal.pone.0263177

**Published:** 2022-01-27

**Authors:** Irene Montiel, Aránzazu Basterra-González, Juan M. Machimbarrena, Jéssica Ortega-Barón, Joaquín González-Cabrera

**Affiliations:** 1 Faculty of Education, Universidad Internacional de La Rioja (UNIR), Logroño, La Rioja, Spain; 2 Faculty of Psychology, Universidad del País Vasco (UPV/EHU), Donostia, Basque Country, Spain; Yale University, UNITED STATES

## Abstract

**Background:**

Loot boxes are an increasingly common type of random microtransaction in videogames. There is some concern about their expansion and entailed risks, especially among adolescents. The actual prevalence of engagement with loot boxes among child and adult population is uncertain, and there is still controversy over the nature of their relationship with problematic gaming and gambling.

**Objectives:**

The aims of this scoping review are to summarize the characteristics and findings of published primary empirical studies about the prevalence of engagement with loot boxes and/or their relationship with problematic gaming and gambling, taking in account the type of sample, time frame and measured variables.

**Methods:**

This study follows the Joanna Briggs Institute’s “Guidance for conducting systematic scoping reviews” and the Preferred Reporting Items for Systematic reviews and Meta-Analyses extension for Scoping Reviews (PRISMA-ScR). Three academic databases provided 299 articles.

**Results:**

Sixteen primary empirical studies met the inclusion criteria for this review. All studies used cross-sectional designs, and most used convenience samples. Twelve study samples were comprised exclusively of gamers, and two were comprised of gamers and/or gamblers. Only six studies included adolescents. The annual prevalence rate of loot box purchases was higher for adult gamers than for adolescents (22.7%–44.2% and 20%–33.9%, respectively), but in studies with general population samples, the opposite was true (24.9% for players aged 13–14 versus 7.8% for adults). In general, the studies suggested a significant positive relationship between engagement with loot boxes and problematic gaming and gambling, but this may be related to the type of engagement (open/purchase/sell), and the characteristics of the study participants (male/female, adolescents/adults, gamers/gamers-gamblers/general population).

**Conclusions:**

This scoping review summarizes the results of recent empirical studies on engagement with loot boxes and discusses how methodological issues may affect their results and interpretation. Recommendations for future research are also provided.

## Introduction

Almost 40% of the world’s population plays videogames [1]. The number of gamers has increased by an average of 6% per year over the past five years, reaching 2.7 billion users by 2020. Revenue in the videogame industry reached $120.1 billion in 2019 and approximately $159.3 billion in 2020, an annual increase of more than 9% [1]. An important source of revenue—and one component of this industry’s success—lies in the incorporation of in-game purchases [2, 3].

Within a videogame, there are several objects (usually different in each videogame) that allow players to gain in-game improvements and advantages and quickly achieve the game’s objectives. Graphic elements that modify the external appearance of the characters by changing their aesthetics, their clothes, or their weapons (so-called *skins*) are also common. These advantages are usually purchased through microtransactions (i.e., a player pays a stipulated price for a specific and known advantage or skin) [4]. *Loot boxes*, also called crates, gachas, cases, or chests, are one specific type of microtransaction in which a random virtual item is purchased [5, 6]. Loot boxes can be purchased using real or virtual in-game money, with the most desired items appearing less frequently [7, 8].

The most established games in the world (e.g., Overwatch, FIFA, Battlefield, Call of Duty, Fortnite, and League of Legends) include loot boxes within their mechanics. The presence of loot boxes in desktop videogames increased 67% between 2010 and 2019 [9], and more than 58% of Google Play and iPhone games contain loot boxes [10]. Among gamers, 78% of adults have purchased at least one loot box [11], and 40.5% of adolescents between the ages of 16 and 18 have purchased one within the past month [12]. One of the most worrisome aspects is that 56% of mobile games containing loot boxes are considered suitable for children aged 7 years or older, and 93% are considered suitable for children at least twelve years of age [10]. There is some evidence about the increasing use of loot boxes, but the real extent of the phenomenon is still uncertain, as are the risks it might entail for both adults and adolescents.

Some authors claim that loot boxes introduce gambling elements into gaming and argue that they may act as a gateway to problematic gambling or that they could aggravate gambling or gaming-related harm [5–7, 13]. Whether the excessive use of loot boxes is best conceptualized under the theoretical framework of problem gambling or problem/excessive gaming is also under debate [4, 13, 14]. A recent meta-analysis found a small but potentially clinically relevant relationship between gambling symptomatology and loot box spending. It was also shown that this association was at least as large as that found between excessive gaming symptoms and loot box spending, suggesting the need for further research [14]. Some longitudinal studies have also pointed out that problematic gaming appears to be an entry behavior to problematic gambling [15]. However, a recent review found little convincing evidence in support of this “gateway hypothesis,” suggesting that further longitudinal research is needed to better understand the links between video gaming and gambling [13].

In light of the above and with the primary purpose of protecting children and adolescents, a legislative debate has been initiated about whether engaging with loot boxes should be considered “gambling.” Some authors emphasize its virtual, uncertain, and random nature, pointing to a relationship with the reward systems present in gambling [16, 17]. Others claim that loot boxes are a clear hybrid between slot machines and collectible card packs [18] and simply have similarities with gambling behaviors [19]. Nielsen and Grabarczyk [20] argue that loot boxes are a specific application of a more general phenomenon called the random reinforcement mechanism, which has been implemented in videogames for decades [17]. These reward mechanisms are also present in slot machines, and their components are a condition of eligibility (a requirement that the player must meet to activate the reward, such as reaching a certain number of points), randomness (e.g., shuffling or throwing dice), and reward (e.g., a new weapon) [20]. These authors argue that only those that are embedded in the broader economy (i.e., those that offer randomized rewards that can be both sold and purchased) can be considered structurally similar to gambling. However, as demonstrated by Drummond et al. [21], virtual items have real-world monetary value to users, irrespective of whether they can be cashed out; therefore, they could be regulated under existing gambling legislation. The relationship between loot boxes and random reward mechanisms raises concerns for adolescents and young people, as these populations are more vulnerable to developing emotional, cognitive, and behavioral problems related to traditional and online gambling [22–25]. Furthermore, these populations may not fully understand the associated underlying mechanisms and reward systems [2, 26]. This makes the issue of loot boxes and their potential similarities to gambling of particular interest when it comes to protecting children and young people, for the same reason that these populations are prohibited from gambling.

The discrepancies in the scientific literature mentioned above can be reflected in the different stances that countries take regarding loot box regulation. For example, Belgium and the Netherlands consider loot boxes to be an illegal form of gambling [27]. In the USA there is no uniform legislation regarding this topic [28] and the United Kingdom have prohibited the use of loot boxes only if their contents can be sold outside of the videogame itself [29]. Other countries, such as China and South Korea, require game developers to disclose the odds of winning the prize [30]. Japan only prohibits some types of loot boxes, such as the “kompu gacha” (boxes with rare rewards that can only be unlocked after purchasing a collection of other loot box items) [31]. Clearly, it is necessary to advance and homogenize scientific/technical criteria to help policymakers standardize such important political and legal guidelines.

Due to the emerging nature of this field, a scoping review is the most appropriate methodological approach for assessing and understanding our research questions. Scoping reviews are particularly useful when a body of literature has not yet been comprehensively reviewed or exhibits a complex or heterogeneous nature [32]. Moreover, scoping reviews allow us to identify further areas for subsequent research and clarify whether a systematic review may be conducted to address more specific questions [33]. Two systematic reviews about loot boxes have recently been published [14, 34], but they focused exclusively on the relationship between loot-box-related spending and problem gaming and/or gambling. These reviews did not summarize the prevalence of engagement with loot boxes or provide separate data for adolescents versus adults. Additionally, many empirical studies that did not appear in these works have recently been published in peer-reviewed journals.

Therefore, we conducted a scoping review of the primary empirical studies on engagement with loot boxes published to date in peer-reviewed journals. Our initial research questions were as follows: 1) How did these primary empirical studies measure engagement with loot boxes? 2) What is the prevalence of engagement with loot boxes among adults and adolescents? 3) Is there a clear positive relationship between engagement with loot boxes and problematic gaming and/or gambling, or does this depend on the study characteristics? We then used this information to offer suggestions regarding measurement issues and propose ideas for meaningful future research.

## Methods

The research and reporting methods of this scoping review are consistent with the Preferred Reporting Items for Systematic reviews and Meta-Analysis extension for Scoping Reviews (PRISMA-ScR) [35], as outlined in the S1 Table. In addition, the present study follows the Joanna Briggs Institute’s “Guidance for conducting systematic scoping reviews” [32, 36], based on earlier work by Arksey and O’Malley [37] and Levac et al. [38], to improve the utility and robustness of the results [32]. The objectives, inclusion criteria, and methods of analysis for this review were specified in advance and documented in a protocol adapted from the protocol template of the International prospective register of systematic reviews (PROSPERO) [39] which is available upon request from the corresponding author.

### Identification of relevant studies

A systematic and comprehensive search was carried out from June 22, 2020 to July 22, 2020 (inclusive of both dates) using the following electronic databases: Google Scholar, Web of Science, and Scopus. The search terms used were “loot boxes” and “loot box.” Because of the scarcity of relevant published literature and the emerging nature of this topic, no limits were placed on the publication dates of the articles. Bibliographic references from qualitative or review studies were reviewed to identify research articles not captured by the electronic search. An additional search was subsequently carried out in the last week of April 2021 to detect new publications.

### Inclusion and exclusion criteria

The inclusion criteria arose from the formulation of the review questions in the PCC (population, concept, context) format [32]. To be included in the scoping review, studies had to meet three eligibility criteria: 1) primary empirical studies with samples of adults and/or adolescents providing data on the prevalence of engagement with loot boxes in videogames and/or data on the relationship between engagement with loot boxes and problematic gaming and/or problematic gambling; 2) published in English or Spanish; and 3) published in peer-reviewed journals. Qualitative studies, theses, reports not published in academic journals, systematic reviews, and meta-analyses were excluded. However, their reference lists were reviewed to locate potentially eligible studies. In cases of pre-prints, authors were contacted to check the status of the studies. One of them provided the published study, which was included in the final selection [16].

### Selection of the studies

**Fig 1:**
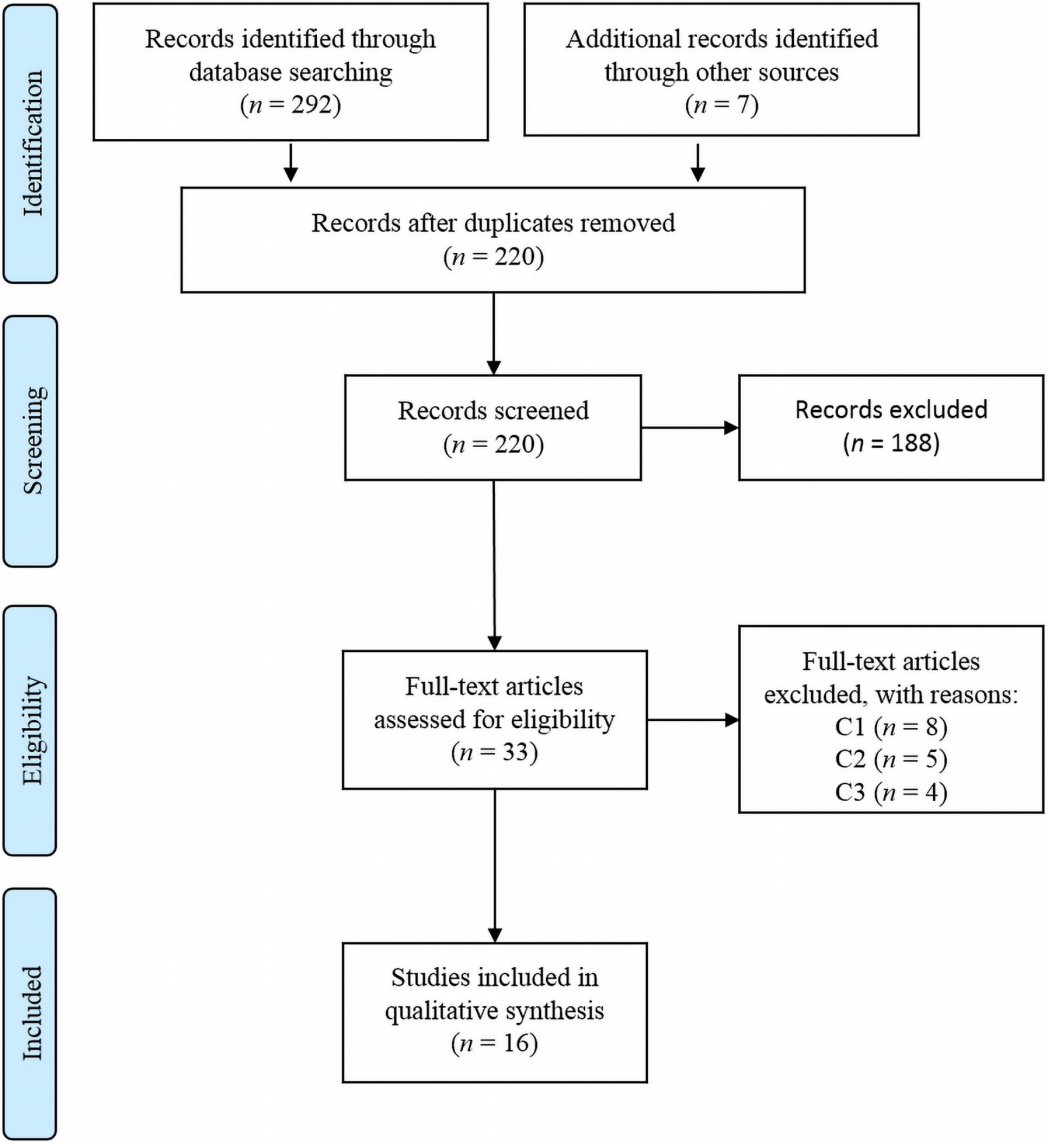
PRISMA diagram of study selection process. *Note*. C1: non-primary empirical studies; C2: studies in a language other than English or Spanish; C3: no peer review.

Fig 1 shows the selection process of studies for this review. In the initial search, 292 manuscripts were identified from the three databases consulted. Seven manuscripts identified through other sources (e.g., reference lists and original authors contacted by email) were added. All references (*n* = 297) were then imported into the Zotero bibliographic manager. Through the bibliographic manager the authors ordered the references and reviewed the titles, abstracts, and digital object identifiers to detect duplicates. Duplicates were eliminated, and a total of 220 items were transferred to the screening phase. To reduce potential bias at this stage of the review, two independent researchers (X.X. and Y.Y.) evaluated all titles and abstracts to pre-select articles that could potentially meet the three eligibility criteria. If an abstract did not allow assessment of study eligibility, the full text was scanned. At this stage, 188 items were removed, leaving a total of 33 pre-selected items. Kappa’s coefficient for agreement between the two researchers in the screening and pre-selection phase was 0.852. After an in-depth reading of the pre-selected articles, 17 were discarded because they did not meet all eligibility criteria. The second researcher reviewed the final selection, and potential disagreements about a study’s final inclusion were solved by a majority consensus among all team researchers. From the last search conducted in April 2021, two articles were added. Finally, 16 studies/articles were included in the qualitative synthesis of this scoping review. Notably, two of the studies [40, 41] included two different samples, as demonstrated in Table 1. This is specified in the narrative synthesis of the results.

**Table 1:** Characteristics of the selected studies (N = 16).

Author (year)	Methods						Outcomes		
	Year and country of data collection	Participants (*n*, sex, age)	Design and recruitment	Objectives	Measured variables/instruments	Loot box def.	Loot box prevalence	Problematic gaming	Problematic gambling
Brooks & Clark (2019)	2018, North America and Canada	(n1) 144 participants ≥ 21 y/o with prior video game play and familiarity with loot boxes; 48.6% ♀; M = 34; SD = 10. (n2) 113 students from The University of British Columbia ≥ 19 y/o with prior video game play and familiarity with loot boxes; 12.1% ♀; M = 21; SD = 2.4.	Cross-sectional data from two convenience samples: one collected via Amazon Mechanical Turk (n1) and the other from an online survey (n2). Compensation of $1.50 USD and a course credit were given to the n1 and n2 participants, respectively.	To test the associations between loot box engagement and gambling behavior, utilizing an exploratory approach.	Use, preferences, virtual item valuation, prioritization of gaming over other activities. **Ever engaged with a loot box (use, purchase), beliefs and behaviors, and monthly loot box spending**. **Risky Loot Box Index (RLI)** Internet Gaming Disorder Scale (IGDS) Problem Gambling Severity Index (PGSI)	Y	Opening loot box in a game: 88.9% (n1) and 94.8% (n2) Buying loot box or key that opens it: 49.3% (n1) and 60.3% (n2) Selling loot box: 27.8% (n1) and 39.7% (n2).	Significant positive association between RLI and IGDS in the two samples.	Significant positive association between scores of RLI and PGSI in the two samples.
DeCamp (2021)	2018/2019, Delaware (USA)	13042 public school students aged 13–16 y/o; 50% ♀, 50% ♂. 2018: (n1) 4678 (8th grade); (n2) 3909 (11th grade). 2019: (n1) 2126 (8th grade) (1531 were gamers); (n2) 2329 (11th grade) (1340 were gamers).	Cross-sectional data from the annual Delaware School Survey (DSS), administered by the University of Delaware Center for Drug and Health Studies, with a **randomly** selected sample.	To examine one dimension of potential similarities between loot boxes/downloadable content and gambling: risk and protective factors.	Traditional forms of gambling. Digital gaming purchases: **number of loot boxes purchased** and times that other downloadable content or in-game items had been purchased in the past year. Others (parental bond, depression/anxiety, victimization, bullying, substance use, school grades, gender, and race/ethnicity).	N	Buy loot box (only 2019 samples) (n1) 24.9% (33.9% of gamers) (n2) 17% (28.3% of gamers).	Does not provide data.	Does not provide data.
Drummond et al. (2020)	2019, New Zealand, Australia, and the USA	1288 respondents (1049 video gamers ≥ 18 y/o). 63.4% ♀, 35.5% ♂; M = 40; SD = 15.4. (17% of the sample met PGSI criteria for gambling problems).	Cross-sectional data from three large cross-sectional, cross-national samples recruited through Qualtrics’ Survey Targeting Tool. Exclusion of participants who endorsed the statement “I once owned a three headed dog”; who indicated that they never played video games; and who were greater than 3.29 standard deviations from the mean on loot box spending (equating to $104.20 USD).	To examine whether problem gambling and problem gaming symptomology made independent contributions to predicting loot box spending.	**Risky Loot Box Index (RLI)** Internet Gaming Disorder Scale (IGDS) Problem Gambling Severity Index (PGSI) **Past month spending on loot boxes**. Others (positive and negative affect, psychological distress).	N	Does not provide data.	Significant positive association between IGD symptomology, loot box spending, and RLI.	Significant positive associations between monthly loot box spending, PGSI, and RLI.
Evren et al. (2021)	2019, Turkey	752 video gamers (eSports players and university students) ≥ 18 y/o. 31% ♀, 69% ♂.	Cross-sectional data from an online survey addressed to members of a database from ESL Turkey Amateur eSports players and Taleworlds Entertainment, users of Turkish gaming forums and university students (rewarded with bonus credit).	To evaluate the relationships of loot box engagement with gender, disordered gaming, using massive multiplayer online role-playing games, and motives for online gaming among young adults.	Weekly gaming time. Motives for online gaming. Internet Gaming Disorder Scale–Short Form (IGDS9-SF) **Last year loot box purchases**.	N	Purchase loot boxes: 22.7%.	Significant positive association between loot box purchase and the severity of IGD symptoms.	Does not provide data.
Ide et al. (2021)	2017, Japan	1615 adolescent gamers aged 14 years (born between September 2002 and August 2004). 37% ♀, 63% ♂. (16.2% of gamers exhibited four or more addictive symptoms in online gaming).	Cross-sectional data from the Tokyo Teen Cohort (TTC) study, an ongoing, prospective, and population-based birth cohort study on 2667 adolescents and their primary caregivers recruited from three municipalities using the resident registers.	To investigate the association between loot box purchasing among adolescents and parents, and problem online gaming in population-based samples.	**Loot box purchase (no time frame specified)**. Gaming Disorder (DSM-5 Internet gaming disorder criteria)	N	Purchase loot boxes: 3.5%.	Significant positive association between loot box purchase and problem online gaming.	Does not provide data.
King et al. (2020)	2018, USA, Australia, Canada, and UK	428 Fortnite gamers ≥ 18 y/o. 6.5% ♀, 91.8% ♂. 57% 18–21 y/o, 85% up to 30 y/o (M = 23.5 y/o, SD = 7.3, median = 21). (14% of the sample met DSM-5 criteria for Internet Gaming Disorder).	Cross-sectional data from online forums related to Fortnite (e.g., Epic Games, Reddit forums).	To investigate gaming motivations and behaviors, as well as online social network influences, in relation to microtransaction spending and gaming disorder (GD) symptoms.	Fortnite social play and influence, Fortnite gaming and expenditure: **Expenditure on loot boxes (daily, weekly, monthly and yearly)**. Gaming Disorder (DSM-5 Internet gaming disorder criteria) Perceptions of Fortnite value (PFV) Others (impulsiveness, gaming-contingent self-worth).	N	Does not provide data.	No significant association between loot box expenditure (yes/no and amount) and GD symptoms.	Does not provide data.
Kristiansen & Severin (2020)	2018, Denmark	1137 adolescents aged 12–17 y/o (995 played video games in the prior 12 months). 50.6% ♀, 49.5% ♂. 43.7% 12–13 y/o; 41% 14–15 y/o; 15.4% 16 y/o.	Cross-sectional data from a survey among a representative gross sample of 5000 Danish adolescents (12–16 y/o) drawn **randomly** from the Danish Civil Registration System. Web survey sent, mainly digitally, to a selected parent of the participant.	To examine loot box engagement patterns and links with problem gambling severity.	Monetary gambling involvement, simulated gambling involvement, self-evaluated exposure from gambling advertisements, and a subsection on gambling related gaming including **loot boxes (past 12 months obtainment, purchase, and sale)**. South Oaks Gambling Screen-Revised for Adolescents (SOGS-RA).	N	Total engagement among gamers 56.1% (40% earned; 20% purchased; 10.6% sold). Obtained loot box: 44.3% 12–13 y/o; 40.7% 14–15 y/o; 42.4% 16–17 y/o. Buy loot box: 20.5% 12–13 y/o; 17.8% 14–15 y/o; 23.5% 16–17 y/o.	Does not provide data.	Significant positive association between loot box purchasing or selling and PG severity (not only obtained), even controlling for demographic factors.
Li et al. (2019)	2018, Unknown	618 video gamers ≥ 18 y/o. 63.7% ♂; M = 27 y/o; SD = 8.9. (20.06% of the sample met DSM-5 criteria for Internet Gaming Disorder). (48.55% of the gamers/gamblers sample met PGSI criteria for gambling problems).	Cross-sectional data from an online survey advertised to the general public via an Internet-based research panel (FindParticipants.com), gaming and research forums (e.g., Reddit), and university students who were members of eSports and video game organizations at a large public university.	To explore the relationships of loot box purchases with both problem video gaming and problem gambling behaviors.	**Past 12 months expenditure on loot boxes**, video games used, and gaming experience. Video gaming and online gambling engagement (frequency). Problem Video Gaming (DSM-5 IGD criteria) Problem Gambling Severity Index (PGSI) Others (mental distress)	N	Buy loot boxes: 44.2% of gamers (66.54% bought daily, 26.84% once a week). 66.1% of gamblers.	Significant positive association between loot box purchase and PVG severity after controlling for gaming engagement. 39.34% of loot box buyers classified as IGD.	Significant positive association between loot box purchase and PG severity. 66.04% of loot box buyers classified as PG.
Macey & Hamari (2019)	2018, USA, UK, Finland, Canada, and 58 other countries	582 video game players who had watched eSports, bet, or bought a loot box in the prior 12 months. 91.9% ♂, 5.5% ♀; 1.9% ≤ 14 y/o; 25.1% 15–17 y/o; 31.3% 18–21 y/o; 16.5% 22–25 y/o; and 23.4% ≥ 26 y/o. (4.5% of the sample met PGSI criteria for gambling problems).	Cross-sectional data from an online survey posted on social media sites, such as Facebook and Reddit, on eSports discussion forums, and on the social media pages of various national eSports associations (only English-language sites). Respondents had the chance to enter a **raffle to win a $50 USD gift card**.	To assess participation rates and demographic characteristics of eSports spectators who gamble and to determine the prevalence of problematic gambling behaviors.	Consumption habits for eSports and gambling behavior (online and offline) (overall engagement). **Purchasing loot boxes (frequency, average weekly hours spent, and average monthly amount spent were combined to calculate *overall engagement in the past year*)**. Problem Gambling Severity Index (PGSI)	N	Buy loot boxes: **46.2%** of those who participate in gambling or gambling-like experiences.	Does not provide data.	Significant positive association between buying loot box and PGSI.
von Meduna et al. (2020)	2018, Germany	6000 Internet users ≥ 18 y/o that had participated in online gambling or Pay2Win gaming. (1508 Pay2Win users: 51.3% ♂).	Cross-sectional data from the larger e-GAMES (Electronic Gam(bl)ing: Multinational Empirical Surveys) with a **representative sample** of the German population of Internet users.	To explore the overlap between gaming and gambling as well as similarities and differences between the countries from a large number of respondents.	Problem Gambling Severity Index (PGSI) Adapted version of PGSI for Pay2Win gaming **Past year purchasing loot boxes (yes/no) and frequency of loot box purchasing**.	N	Buy loot box: 38.9% of Pay2Win users 9.8% of all people completing the survey (including non-Pay2Win users).	68.9% of loot boxes purchasers are problem gamers, but there are mixed results in the regression models.	Significant positive association between loot box participation, purchasing frequency, and PGSI, but with mixed results. 45.9% of loot boxes purchasers are problem gamblers.
Wardle & Zendle (2021)	2019, Great Britain	3549 participants aged 16–24 y/o who had not taken part in any other YouGov study on gambling in the past year. 51.3% ♂, 48.7% ♀; 33.4% 16–18 y/o; 31% 19–21 y/o; 35.6% 22–24 y/o. (42.5% had gambled on any activity in the past year and 3.7% experienced problem gambling).	Cross-sectional data from Emerging Adults Gambling Survey. Participants were drawn from YouGov’s online panel of over 1 million people living in Britain. Emails were sent by YouGov to a random selection of their panel members, stratified by region. Data were weighted to reflect the age, sex, and regional profile of Great Britain.	To examine the relationship between the purchase of loot boxes, gambling behavior, and problem gambling among young people ages 16–24.	Problem Gambling Severity Index (PGSI) A suite of questions adapted from the UK Gambling Commission’s Youth Gambling Survey asked whether participants played video games in the last year. **Last year use of their own money to open loot boxes**. Other measures (impulsivity, ethnicity, age, employee).	N	Total loot box purchase: 12.1%: 38.9% (16–18 y/o), 29.4% (19–21 y/o), 31.8% (22–24 y/o).	Does not provide data.	Significant positive association between loot box purchase and PG after controlling for other variables. 16.9% of loot box purchasers were problem gamblers.
Zendle & Cairns (2018)	2018, USA, UK, Canada	7422 gamers ≥ 18 y/o 9% ♀, 88% ♂. 48% 18–24 y/o; 27.8% 25–29 y/o; 14.3% 30–34 y/o; 6% 35–39 y/o; 3.5% > 45 y/o.	Cross-sectional data from an online survey with a self-selected sample through Reddit and subreddits (online bulletin board).	To measure both how much these individuals spent on loot boxes and the links between purchasing loot boxes and problem gambling.	Problem Gambling Severity Index (PGSI) **Ever purchased loot boxes (Y/N)**. **Monthly loot box expenditure**. Other in-game microtransaction spending.	N	Buy loot box: 78%.	Does not provide data.	Significant positive association between PGSI and monthly loot box spending.
Zendle & Cairns (2019)	2018, USA	1172 regular gamers of any of these ten games (≥ 18 y/o): Player Unknown’s Battlegrounds, League of Legends, Hearthstone, Overwatch, Counter-Strike: GO, FIFA 18, Rocket League, DOTA 2, Team Fortress 2, and Rainbow Six Siege of Tom Clancy. 31% ♀, 64% ♂. 20% 18–24 y/o; 29% 25–29 y/o; 12% 35–39 y/o; 12% > 40 y/o.	Cross-sectional data from an online survey through Amazon Mechanical Turk. The recruitment message **did not** specifically **mention loot boxes**.	To measure problem gambling and loot box spending in a sample of gamers from the USA.	Problem Gambling Severity Index (PGSI) **Past month loot box expenditure**. Other spending habits.	N	Does not provide data.	Does not provide data.	Significant positive association between PGSI and past month loot box spending.
Zendle et al. (2019)	2018 UK, Canada, Australia, New Zealand, and some European countries	1155 adolescent gamers aged 16–18 y/o. 9% ♀, 88% ♂. 26.4% 16 y/o; 26.6% 17 y/o; and 47% 18 y/o.	Cross-sectional data from an online survey through Reddit and subreddits (online bulletin board) or specialist interest bulletin boards for games that featured loot boxes.	To measure the relationship between loot box spending and problem gambling.	Canadian Adolescent Gambling Inventory’s (CAGI) Problem Gambling subscale. Past month loot boxes opening and purchase (Y/N). **Monthly loot box expenditure**. Features of loot boxes (in-game currency, contents, etc.) and motivation. Other in-game microtransaction spending.	N	Pay for a loot box: 40.5%.	Does not provide data.	Significant positive association between monthly loot box spending and PG.
Zendle (2020)	2019, UK	1081 participants ≥ 18 y/o; 549 ♀, 526 ♂. 190 (18–27 y/o), 176 (28–37 y/o), 203 (38–47 y/o), 184 (48–57 y/o), 328 (> 58 y/o).	Cross-sectional. Online survey by quota sampling to **represent the UK population** in terms of sex, age, and ethnicity. Sample recruited by Prolific Academic, an online panel provider that **remunerated the participants**.	To estimate the prevalence of loot box spending, eSports betting, real-money gaming, watching videos, etc., and their links with problem gambling and disordered gaming.	Frequency of engagement in eleven traditional forms of gambling, gaming-related forms of gambling and gambling-like behaviors in the past 12 months (eSports betting, **loot box spending, frequency of loot box purchase**, watching loot box openings online, etc.). Problem Gambling Severity Index (PGSI) Internet Gaming Disorder Scale (IGDS)	Y	Engaged in loot box spending: 7.8%.	Significant positive association between purchasing frequency and IGDS.	Significant positive association between purchasing frequency and PGSI.
Zendle et al. (2020)	2018, Unknown	1200 participants, ≥ 18 y/o players 37.1% ♀, 60.8% ♂. 19.8% 18–24 y/o; 27.3% 25–29 y/o; 25.2% 30–34 y/o; 13.3% 35–39 y/o, and 14.4% > 40 y/o. (17.7% of the sample met PGSI criteria for gambling problems).	Cross-sectional data from an online survey through Amazon Mechanical Turk workers **without mentioning loot boxes**.	To determine if loot boxes features strengthen the link between loot box spending and problem gambling.	Problem Gambling Severity Index (PGSI) **Past month loot box purchasing** **Past month expenditure in loot boxes** Past month earnings selling loot boxes. Others (near-misses, in-game currency, crate and key mechanisms, exclusive items).	N	Engaged in unpaid openings: 37.6% Pay to open loot box: 62.4%.	Does not provide data.	Significant positive associations between PGSI and loot box spending and between paying for loot boxes and PGSI (higher than free loot boxes), even when cashing out is not possible.

*Note*. M = mean; SD = standard deviation; Y/o = years; ♂ = boys; ♀ = girls; Y = Yes; N = No; IGD = Internet Gaming Disorder; PGSI = Problem Gambling Severity Index; SOGS-RA = South Oaks Gambling Scale–Revised Adolescents; RLI = Risky Loot Box Index; GD = Gaming Disorder; PG = Problem Gambling; PVG = Problem Video Gaming; DG = Disordered Gaming; Bold text = relevant characteristics.

### Data extraction

Table 1 provides a summary of all information extracted from the 16 studies included in the final review. A standardized extraction sheet (Microsoft Excel) containing the following variables was developed: authors and publication year, country and year of data collection, sample size and age and sex of participants, design and recruitment, objectives, measurement instruments, and outcomes of the studies relevant to the aims of this scoping review (i.e., rate of engagement with loot boxes and results concerning the presence or absence of a statistically significant relationship between engagement with loot boxes and problematic gaming/gambling). Table 2 shows the studies’ loot box engagement rates by age group (adolescents versus adults), time frame, and behavior assessed (open/purchase/sell). After piloting the extraction sheet within the review team, data extraction was performed by one team researcher and cross-checked by another team member for accuracy.

**Table 2:** The overall prevalence of engagement with loot boxes according to time frame, age group, and action taken (*N* = 16).

Time frames	*n*	Prevalence range				
		Open (%)	*n*	Purchase (%)	*n*	Sell (%)
**Lifetime (*n* = 2)**						
Adolescents	0	-		-		-
Adults	1	88.9^1^–94.8^1^	2	49.3^1^–78^1^	1	27.8^1^–39.7^1^
**Past year (*n* = 9)**						
Overall (mixed)	0	-	2	12.1^2^–46.2^3^	0	-
Adolescents	1	40.7^1^–44.3^1^	2	20^1^–33.9^1^	1	10.6^1^
				17^2^–24.9^2^		
Adults	0	-	4	22.7^1^–44.2^1^	0	-
				7.8^2^–9.8^2^		
				66.1^3^		
**Monthly (*n* = 2)**						
Adolescents	0	-	1	40.5^1^	0	-
Adults	0	37.6^1^	1	62.4^1^	0	-

*Note*:

^1^Gamer sample;

^2^General population sample (gamers, non-gamers, gamblers, and non-gamblers);

^3^Gamer/gambler sample.

## Results

### Characteristics of the studies

Of the 16 selected studies (Table 1), nine were carried out with samples from a single place of origin. Four of these studies used samples limited to Europe (two from the United Kingdom [42, 43], one from Denmark [2], and one from Germany [16]. Three studies were conducted using samples from the USA [6, 40, 41], and two were conducted using samples from Asia [44, 45]. For two studies, samples were collected through the Internet, and the origin of the participants was unknown [3, 46]. The other five studies obtained samples from several countries [5, 11, 12, 47, 48]. The study by Macey et al. [48] is noteworthy for its diversity, as it included a sample from 61 countries. All studies were carried out between 2017 and 2019 with a cross-sectional design and were published in peer-reviewed journals between 2018 and 2021.

The sample sizes were heterogeneous, ranging from 113 participants [40] to 13,042 [41]. The ages of study participants ranged from 12 years [2] to 58 and older [43]. Of the 16 studies selected, only four had samples made up exclusively of adolescents between 12 and 18 years of age [2, 12, 41, 45], and ten had samples made up of only adults over 18 years of age [3, 5, 6, 11, 16, 40, 43, 44, 46, 47]. The remaining two studies had mixed samples composed of both adolescents and adults [42, 48].

Five samples were obtained from the data of four larger national studies [2, 16, 41, 45] conducted through population-based sampling: one representative sample of the adolescent population from Denmark [2], two from Delaware [41], one from Tokyo [45], and one representative sample of the German population of adult Internet users [16]. Additionally, one study used participants recruited from an online survey by quota sampling to represent the UK adult population [43]. Another study used an online panel survey in which data were weighted to reflect the age, sex, and regional profile of the population between 16 and 24 years old in Great Britain [42]. Of these seven samples, four included gamers, non-gamers, gamblers, and non-gamblers and similar proportions of male and female respondents (general population samples)—two samples of Delawarean adolescents [41], one of British adults [43], and one of British youth between 16 and 24 years old [42]. The other three samples required fulfillment of some inclusion criteria (e.g., being video gamers [2, 45] or being gamblers or Pay2Win users [16]).

Ten studies used convenience sampling procedures in which participants were recruited through various online platforms, such as Reddit [11, 12, 48], Amazon Mechanical Turk [6, 40, 46], Findparticipants.com [3], or gaming forums [3, 44, 47]. In four of these ten studies, participants received an economic or academic reward [40, 43, 44, 48]. Two of the studies blinded the aims of the research and did not mention loot boxes in their recruitment messages [6, 46]. All of these study samples required fulfillment of some inclusion criteria, such as being video gamers [3, 5, 11, 12, 40, 44, 46]; video gamers who had watched eSports, bet, or bought loot boxes [48]; or gamers of specific videogames [6], such as Fortnite [47].

In summary, twelve studies in this scoping review used samples composed exclusively of gamers [2, 3, 5, 6, 11, 12, 40, 44–47]. Two were composed of gamers and/or gamblers [16, 48]. In most of these samples, there was a higher proportion of male respondents than females [6, 11, 12, 40, 44–48]. However, one sample had a higher proportion of female respondents [5]), and three others had similar proportions of males and females [2, 16, 40]. Furthermore, of the studies involving gamer samples, two included a high proportion of people with gaming problems [3, 45] and four included a high proportion of people who presented gambling problems [3, 5, 46, 48].

#### Operationalization and measurement of loot box engagement

All included studies provided homogeneous definitions with regard to the characteristics of the construct in question such as virtuality, randomness, and monetary payment. Some authors nuanced the definitions, supplementing them with terms such as “digital reward,” “bet,” “uncertain value,” or “in-game or real-world currency” [3, 5, 16, 42, 43, 48]. Only two studies provided a specific definition of loot boxes before asking about their use [40, 43].

Regarding the operationalization of the use of loot boxes, not all studies asked about the same behaviors or types of engagement. Thirteen evaluated the purchase of loot boxes [2, 3, 11, 12, 16, 40–46, 48], two evaluated the sale or “cash-out” [2, 46], and two also measured the opening of a loot box in general, with or without payment [2, 40]. For this purpose, sociodemographic questions designed ad hoc (such as “Have you bought a loot box in the last year?” [2]) were used. In eleven of the studies, the questions prompted a dichotomic response of “yes” or “no” [2, 11, 12, 16, 40, 42, 43, 46, 48]; one asked for the number of loot boxes purchased [41]; and another calculated overall engagement from different variables, such as frequency of purchasing or time spent [48]. Six of the 16 selected studies asked participants about their expenditure on loot boxes [3, 5, 6, 12, 46, 47]; five of these studies used an open-answer format to indicate the amount spent [5, 6, 12, 46, 47], and the other one used a closed-answer spending bracket format (ranging from < $1 to > $300 in the past month) [11].

Regarding the time frames analyzed in the studies, two asked about the participants’ lifetimes [11, 40], nine focused on the past year [2, 3, 16, 41–44, 48], four examined the past month [5, 6, 12, 46], one asked about different time frames (daily, weekly, monthly, and yearly) [47], and one did not specify a time frame [45].

Brooks and Clark [40] designed an instrument to evaluate risky behaviors surrounding the use of loot boxes that may become problematic. Their “Risky Loot Box Index” (RLI) consists of five items associated with three dimensions: cognitive concern about the use of loot boxes, impulsive use, and chasing losses. This instrument has demonstrated adequate reliability (α = .864), although its internal validity is limited.

#### Prevalence of loot boxes

There was great heterogeneity among the data regarding loot box engagement prevalence from the 16 selected studies. These data were presented in terms of whether or not loot boxes were purchased, obtained/opened (without specifying payment), or sold; the type of sample evaluated (adolescents or adults over 18 years of age); and the time frame analyzed (lifetime, annual, or monthly) (Table 2). Notably, analysis of the behaviors surrounding loot box purchases revealed that none of the studies differentiated between direct purchases with real money (legal tender) and purchases using virtual in-game/ecosystem currency (which must have been previously purchased with legal tender). All studies took a general approach to the purchase of loot boxes without qualifying this aspect.

*In adolescents*. Of the 16 studies analyzed, four exclusively studied adolescents (12 to 18 years of age) [2, 12, 41, 45]. The first study used two different representative samples of Delawarean adolescents (8^th^ grade and 11^th^ grade) to analyze the annual prevalence of loot box purchases. This rate ranged from 17% for ages 16–17 (*n* = 2,329) to 24.9% for ages 13–14 (*n* = 2,126) (28.3% and 33.9%, respectively, in the gamer samples) [41]. The second study, with a representative sample of 1137 Danish adolescents between 12 and 17 years of age, revealed that the annual prevalence rate of loot box purchasing among gamers (*n* = 995) was 20% [2]. The third study analyzed the monthly prevalence of loot box purchases among 1155 adolescent gamers (aged 16 to 18 years) and reported it to be 40.5% [12]. The fourth study revealed a purchase prevalence of 3.5% in a sample of 1615 adolescent Japanese gamers (14 years old), but this study did not specify the time frame analyzed [45].

Only one study on adolescents analyzed data regarding other forms of engagement with loot boxes. Among gamers from a representative sample of Danish adolescents between 12 and 17 years, there was a higher annual rate of obtaining a loot box (with or without paying, 40.7%) than of purchasing one (paying, 20.5%) [2].

*In adults*. Ten of the 16 studies used adult samples [3, 5, 6, 11, 16, 40, 43, 44, 46, 47]. Of these studies, six reported an annual prevalence rate of loot box purchases. The rates ranged from 7.8% (in a representative sample of adults from the United Kingdom [43]) to 9.8% (in a representative sample of German adult Internet users [16]) to 44.2% (in a sample of gamers) and 66.1% (in a sample of gamers who also gambled [3]). Furthermore, in a sample comprised of gamers, one study determined that the monthly prevalence of loot box purchases was 62.4% [46]. Two studies examined this prevalence throughout participants’ lifetimes, revealing rates of 49.3% [40] and 78% [11]. One study included two different convenience samples of adult gamers [40]. It addressed the lifetime prevalence of different loot-box-related behaviors, such as 1) participating in games containing a loot box, 2) opening a loot box, 3) buying a loot box, and 4) selling a loot box. The prevalence rate of opening a loot box was 88.9% in the first sample (*n* = 144) and 94.8% in the second sample (*n* = 113). In the case of loot box purchases, the rates were 49.3% in the first sample and 60.3% in the second sample. In the case of selling loot boxes, the rates were 27.8% in the first sample and 39.7% in the second sample. The rest of the studies involving adults (*n* = 3) did not provide data on the prevalence of engagement with loot boxes; they only measured loot box expenditure [5, 6, 47].

*In mixed samples of adults and adolescents*. Two studies examined mixed samples of adults and adolescents [42, 48]. One used a convenience sample and determined that 46.2% of gamers who also gambled or watched eSports (*n* = 582) had bought a loot box in the prior year [48]. The other study demonstrated an annual loot box purchase prevalence of 12.1% using an online panel survey of 3,549 British participants between 16 and 24 years, with younger people being the biggest purchasers (38.9% were between 16 and 18 years of age) [42].

#### Association between engagement with loot boxes and problematic gaming and gambling

Eight of the 16 studies analyzed the relationship between engagement with loot boxes and problematic gaming. Seven analyzed adult samples [3, 5, 16, 40, 43, 44, 47]. One sample was representative of the German adult population of Internet users [16], and another used quota sampling to represent the UK adult population in terms of sex, age, and ethnicity [43]. One of the eight studies used a sample of adolescent gamers from a population-based birth cohort study in Japan [45]. Six of the eight studies revealed a positive, statistically significant relationship between the two constructs [3, 5, 40, 43–45]. However, one study did not find this relationship [47], and another showed mixed results [16]. Of those six studies, five reported a positive relationship between loot box purchases and problematic gaming as measured by the Internet Gaming Disorder Scale (IGDS [43, 49]), the Internet Gaming Disorder Scale–Short Form (IGDS9-SF; [44, 50]), and the DSM-5 Internet Gaming Disorder criteria (IGD; [3, 23, 43, 45]). Two studies reported a positive relationship between RLI score and higher scores for problematic gaming as measured by the IGDS [5, 40]. A study by von Meduna et al. [16] indicated that 68.9% of loot boxes purchasers were problem gamers according to an adapted Problem Gambling Severity Index (PGSI) score for Pay2Win gaming, but this study highlighted mixed results in the regression models; namely, the authors observed that having problems with Pay2Win gaming was significantly associated with loot box purchasing (yes/no) in all three tested models but had no significant effect on the frequency of loot box purchasing. Drummond et al. [5] observed a positive correlation between loot box spending and higher scores for disordered gaming as measured by the IGDS. Only one study, carried out in a sample of adult Fortnite gamers, did not find a positive relationship between loot box expenditure (yes/no and amount) and IGD symptomatology as measured by the DSM-5 criteria [47]. Notably, some studies found the association between loot box engagement and problem gaming to be associated with personal variables, such as the participants’ sex. For example, Ide et al. [45] observed in a sample of Japanese adolescents aged 14 that the likelihood of presenting problem online gaming was significantly higher in adolescent female gamers who purchased loot boxes (OR 6.73, 95% CI 2.42–18.72) than in male gamers who purchased loot boxes (OR 2.88, 95% CI 1.51–5.51).

Furthermore, 12 of the 16 selected studies analyzed the relationship between engagement with loot boxes and problematic gambling. They all indicated a positive association between the two constructs [2, 3, 5, 6, 11, 12, 16, 40, 42, 43, 46, 48]. Two of these 12 studies were carried out using adolescent samples [2, 12], two with mixed samples of adolescents and adults [42, 48], and the remaining eight with adult samples. Only four of these 12 studies used representative samples of their populations of interest: one used a representative sample of the German adult population of Internet users [16]; one used quota sampling to represent the UK adult population in terms of sex, age, and ethnicity [43]; one used a representative sample of 1137 Danish adolescents between 12 and 17 years of age [2]; and one used an online panel survey of 3,549 British youth between the ages of 16 and 24 [42]. Seven of these 12 studies reported a positive relationship between the purchase of loot boxes and problematic gambling as measured by the PGSI [51] in adults [3, 16, 42, 43, 46, 48] and the South Oaks Gambling Screen–Revised for Adolescents (SOGS-RA; [2, 52]) in adolescents. Two studies found a positive relationship between RLI score and higher scores for problematic gambling as measured by the PGSI [5, 40]. Five studies found a positive correlation between loot box expenditure and higher scores for problem gambling as measured by the PGSI in adults [5, 6, 11, 46] and the Canadian Adolescent Gambling Inventory (CAGI; [53]) in adolescents [12]. The study by von Meduna et al. [16] indicated that 45.9% of loot box purchasers were problem gamblers according to the PGSI, but this study highlighted mixed results in the regression models. Namely, the authors observed that being a problem gambler yields a significant positive effect on loot box purchasing (yes/no) and loot box purchasing frequency in some of the regression models but not in others.

Some studies found that the association between engagement with loot boxes and problem gambling was associated with variables such as sex, age, and type and level of loot box engagement. For example, Kristiansen & Severin [2] found, in a representative sample of Danish adolescents, that this relationship was markedly stronger for females than for males (females: local γ = 0.777, p = 0.043; males: local γ = 0.541, p < 0.01) and for the two older groups (12–13 yrs.: local γ = 0.294, p = 0.322; 14–15 yrs.: local γ = 0.779, p < 0.01; 16–17 yrs.: local γ = 0.565, p < 0.1). They also found that this link was dependent upon the level of engagement. They found that the proportions of at-risk and problem gamblers were higher among those who had purchased or sold items from a loot box than among those who had obtained a free loot box. Similarly, Zendle et al. [46] observed that the positive association between engagement with loot boxes and PGSI scores in a convenience sample of adult gamers was higher when participants paid for the loot boxes than when they did not.

## Discussion

To date, numerous studies about loot boxes have been published. However, to our knowledge, no work has summarized the prevalence rates of different forms of engagement among adolescents and adults or synthesized the most recent empirical results about the relationships between these forms of engagement with loot boxes and problematic gaming and problematic gambling, taking into account methodological aspects of the existing studies. The present scoping review aimed to concurrently examine these aspects of the existing literature.

With regard to methods of measuring engagement with loot boxes in the existing primary empirical studies, we observed strong agreement concerning the characteristics of loot boxes, such as the virtuality of the objects, randomness, and monetary payment (with real currency or in-game currency earned through gaming time). Nevertheless, very few studies were sufficiently consistent in their methods to allow comparison of their results. Each study measured different aspects of loot boxes, such as the opening, purchase (yes/no), frequency, number of loot boxes and/or loot box expenditure, and use across various time frames, making it difficult and inappropriate to compare results between studies. Additionally, as far as we know, there is only one validated instrument that evaluates risky behaviors surrounding the use of loot boxes—the Risky Loot Box Index [40]. However, it would be advisable to carry out more robust analyses of this tool’s psychometric properties.

The heterogeneity of the methods used prevent us from truly understanding the magnitude of the phenomenon. Taking all data extracted from the studies without differentiating among time frames or loot box behaviors (purchase, opening, etc.), prevalence rates among adolescents varied between 3.5% [45] and 44.3% [2], and those among adults varied between 7.8% [43] and 94.8% [40]; however, these data are incomparable among them. The annual purchase prevalence among adolescent gamers varied between 20% [2] and 33.9% [41]. Among adult gamers over the age of 18, the annual purchase prevalence varied between 22.7% [44] and 44.2% [48], but the range was greater among gamers who also gambled (46.2%–66%) [3, 48]. As we can see, among gamers, annual loot box purchase prevalence was higher in adults than in adolescents. This makes sense considering that minors are subjected to parental control, enjoy less freedom, and have less purchasing power. However, in studies that used general population samples, the average annual purchase prevalence among adolescents aged 13–14 years was higher than that among participants aged 16–24 years and that among adults (24.9%, 12.1%, and 7.8%, respectively) [41–43]. This suggests that loot box usage is prevalent among adolescents, regardless of whether they recognized themselves as gamers or not. In addition, these results are in line with those of other works suggesting that age is negatively correlated with this behavior [11, 16, 48, 54], as younger people, in general, are more tech savvy and open to trying something new. However, once an individual comes into contact with a loot box, age is not a moderating variable that reduces the frequency of loot box usage [16]. In any case, it is expected that engagement with loot boxes among adolescents will continue growing in the absence of relevant interventions or legal measures.

Additionally, the differences in results across the existing studies may be associated with cultural and legal variables specific to each country. For example, the 3.5% loot box purchase rate in Tokyo [45] and the 7.8% rate in the UK [43] are far from the 38.9% rate in Germany [16]. This could be a result of differences in the legal status of loot boxes in these countries. In Germany, there are legal barriers to regulating loot boxes as gambling [19], whilst in the UK, loot boxes are covered by the gambling legislation, and in Japan, some types of loot boxes are prohibited [31].

Concerning the last of our research questions, there appears to be a positive relationship between loot boxes and problematic gaming and gambling. However, further research is needed, and sampling bias must be considered when interpreting the results of the existing studies. It is worth mentioning that only eight of the studies analyzed the relationship between loot boxes and problematic gaming, and only one of them used an adolescent sample, despite the facts that 1) access to loot boxes occurs exclusively through videogames and 2) adolescents are heavy videogame users [55]. In general, a positive association between the constructs was observed [3, 5, 16, 40, 43–45, 47]. However, this was primarily true for adult samples and, in some cases, for an overrepresentation of participants classified as problem gamers [3, 5, 45, 47]. The only study that did not find an association between loot boxes and gaming disorder symptoms was based exclusively on Fortnite [47], which may have influenced its results.

In addition, the differentiated effect of opening free loot boxes (versus paid loot boxes) should be considered. The increase in gaming time required to obtain loot boxes might suggest a possible link with the development of problematic gaming, but none of the studies reviewed addressed this. However, some studies in our scoping review examined a similar line of research, pointing out that paid loot boxes are more strongly associated with problem gambling than unpaid loot boxes. For example, Kristiansen and Severin [2] stressed that there was only a relationship between loot boxes and problem gambling when it came to buying or selling loot boxes, not when they were obtained for free. Furthermore, Zendle et al. [48] observed a stronger association between loot boxes and problem gambling when a price was paid than when they were obtained for free.

As far as the relationship with gambling is concerned, it seems reasonable to infer that the gambling nuance attached to loot boxes favors the relationship between loot box purchases and problematic gambling [3, 16]. This has been corroborated by the existing studies to date in both adult and adolescent samples and in a recent secondary analysis of loot box expenditure data [34]. However, caution is necessary when interpreting such results. The scientific literature is still scarce, and an overrepresentation of adults and people with gambling problems [3, 5, 46, 48] was observed in the samples of some studies included in this scoping review. This is also true for the aforementioned secondary analysis by Close et al. [34]. Such a limitation may influence the results and limits generalization. On the other hand, although few studies have simultaneously analyzed the relationship between loot boxes, problematic gaming, and problematic gambling [e.g., 3, 5, 14, 38, 41], their results point to a statistically significant relationship between purchase of (or expenditure toward) loot boxes and the two problems, in line with the results of a meta-analysis by Garea et al. [14]. In short, it can be concluded that loot boxes are linked with problems related to both to gaming and gambling; however, to learn more about these relationships, longitudinal studies and representative samples are necessary. All studies in the present review were cross-sectional in nature, and most of them were of limited representativeness. Therefore, they do not allow establishment of causal effects between variables or extrapolation of their results to the general population.

### Recommendations for research

In view of the results obtained in this scoping review, some recommendations can be made for further research. First, future studies should standardize measurements of engagement with loot boxes to allow for comparisons between studies. This will also favor overall comprehension of the construct and facilitate understanding of the real magnitude of this phenomenon. For example, providing study participants with an established definition of loot boxes (perhaps with some examples and pictures) before asking questions about usage habits might be a good practice. A potential starting point for a definition that includes all necessary elements of a loot box as proposed by different authors [16–20] might be the following: “a loot box is a virtual object (such as a chest, a key, envelope, etc.) within a videogame that offers random contents (such as equipment, weapons, characters, etc.) within the game itself in exchange for an amount of money (either real money paid directly or real money that has been transformed into a virtual currency within the videogame or game ecosystem).”

Secondly, regarding the time frame, we recommend including several factors that can be valuable. Currently, most existing literature has evaluated loot box engagement during the prior year and whilst continue doing so, would facilitate comparison with the current literature, it might also be of interest to know whether this behavior has taken place in the last year (yes/no), frequency which with it happens, (rarely, once or twice, often, or many times in the last year) and the average number of loot boxes purchased/opened/sold or average expenditure. However, when using continuous measures, it will be desirable to ask about shorter periods (e.g., the previous month), since participants’ responses regarding the annual or lifetime timeframe may contain more biases [56].

Thirdly, it will also be relevant to carry out investigations in which there is a clear distinction between the opening of free loot boxes and the opening of paid loot boxes. This will allow analysis of their differing effects on problematic gaming and/or gambling. It may also be relevant to perform analysis as a function of the videogames in which loot boxes are used or of the type of loot box (e.g., purely cosmetic or central to the game). In addition, it should be asked whether these purchases are made directly with real money or with virtual currency through a game/platform ecosystem (after payment with real money).

Furthermore, facilitators of future population-based studies should include similar proportions of males and females as well as wider age ranges, provide aggregated and disaggregated data for each subsample, discuss their results with similar subsamples from other studies, and take steps to avoid limitations to interpretation and generalization. Additionally, in view of the existing data [57–59], it may be noteworthy to increase the proportions of girls in studies about loot box engagement. It would also be advisable to differentiate between analyses with clinical and non-clinical samples and to use clinical and specific instruments for the online context, such as the Online Gambling Disorder Questionnaire [60]. Alternatively, when using the PGSI, it might be advisable to explicitly state that the study is not assessing a clinical problem but rather its more social aspects [61]. Finally, additional instrumental studies are needed to assess the construct of problematic loot box usage as it relates to gaming and gambling problems, both in general and clinical populations.

The present scoping review does have some limitations that should be mentioned. First, the review included a small number of studies, which may be due to the novelty of the subject matter and the inclusion criteria chosen. For example, relevant studies not published in peer-reviewed journals (e.g., theses, unpublished dissertations, reports) were left out of this review. Secondly, the heterogeneity and limited representativeness of some of the samples hindered the process of comparison between studies, which may lead to biases in their interpretation, and their results cannot be extrapolated to the general population. For this reason, we presented the age of the study sample, the time frame, and the behavior assessed in each study as separate factors. Thirdly, all studies included in this review were cross-sectional, which must be considered when interpreting results on the relationship between loot boxes and problematic gaming and/or gambling. Finally, caution is advised because the scientific literature is still scarce and very few studies differentiated between types of engagement (opening, purchasing, or selling) in their analyses.

## Conclusion

In summary, this study contributes to a better understanding of engagement with loot boxes in videogames. First, this study confirms that the use of loot boxes is prevalent among both adults and adolescents. The results also suggest that the purchase of loot boxes is a frequent practice among minors. However, data on this prevalence are heterogeneous, primarily due to methodological differences (e.g., the operationalization of engagement with loot boxes, the samples’ characteristics, and various cultural or legal contexts). This makes the results incomparable across studies and countries. Second, available data suggest a significant relationship between engagement with loot boxes and gambling and gaming problems. Finally, it is necessary for future studies to be conducted in a manner that allows comparability—for example, using common definitions, similar time frames, and similar assessment instruments (particularly to assess the relationship with problematic gaming and gambling). Effective policies for preventing problematic gaming and/or gambling must be based on scientific evidence; thus, a valid and thorough understanding of the magnitude of this phenomenon is essential.

## Supporting information

S1 Table PRISMA-ScR checklist. (DOCX)
